# Inappropriate Medications Use and Polypharmacy among Older Adults with Anxiety Disorder

**DOI:** 10.3390/jcm12134195

**Published:** 2023-06-21

**Authors:** Monira Alwhaibi

**Affiliations:** 1 Medication Safety Research Chair, College of Pharmacy, King Saud University, Riyadh 11149, Saudi Arabia; malwhaibi@ksu.edu.sa; 2 Department of Clinical Pharmacy, College of Pharmacy, King Saud University, Riyadh 11149, Saudi Arabia

**Keywords:** elderly, anxiety, Beers criteria, inappropriate prescribing

## Abstract

Background: Elderly with mental health conditions usually use multiple medications, which predisposes them to inappropriate use of medications, which is defined as medications that should be avoided due to their risk; this outweighs their benefit given that safer alternatives are available. This study aimed to examine potentially inappropriate medication use among older patients with anxiety disorder. Methods: This study used a cross-sectional retrospective study design using twelve months of data extracted from the Electronic Health Record (EHR) database for older adults diagnosed with anxiety disorder and treated in the ambulatory care setting. Potentially inappropriate medications (PIMs) use was evaluated using the 2019 Beers criteria. Descriptive statistics were used to describe the sample. Pearson’s chi-square tests (for categorical variables) and t-tests (for continuous variables) were utilized to measure the differences in independent variables between patients with and without PIMs. Binary logistic regression was used to examine the associations between PIMs use and identify potential factors for PIMs use among older adults with anxiety disorder. Analyses were performed using the Statistical Analysis Software version 9.4 (SAS^®^ 9.4). Results: The study sample includes 371 older adults (age ≥ 65 years) with anxiety disorder; their average age was (72.1 ± 5.8) years. PIMs use was highly prevalent among older adults with anxiety (66.6%). About 35.6% of the study sample used one PIM, 22.6% used two PIMs, and 8.4% used three PIMs. The most frequently prescribed PIMs were NSAIDs and gastrointestinal agents. The adjusted regression analysis found that PIMs use was less likely among men than women. In addition, PIMs use was more likely among women with diabetes, cancer, and polypharmacy. Conclusions: Future studies on strategies and interventions rationing PIMs use in older adults with anxiety disorder are necessary given the high prevalence of PIMs and polypharmacy within this population.

## 1. Background

Anxiety disorder is a mental health condition that can lead to considerable dysfunction affecting physical, mental, and social functioning [1]. It is a highly prevalent mental health condition; globally, up to 25% of adults suffer from anxiety disorder [2,3]. It is less prevalent among older adults than younger adults; up to 11% of older adults suffer from anxiety disorder [4,5,6]. In Saudi Arabia, it is predicted that the prevalence of anxiety and other long-term problems with physical and mental health related to aging will increase [7]. Anxiety frequently co-occurs with other chronic diseases in older persons, which may be associated with increasing polypharmacy and inappropriate medications use (PIMs) to effectively manage these co-occurring chronic health disorders. PIMs use has been defined as “medications that should be avoided due to their risk which outweighs their benefit and when there are equally or more effective but lower risk alternatives available” [8]. PIMs use among older patients has negatively affected health outcomes. It increases the risk of drug-related problems, hospitalization, disability, and other adverse health outcomes by two to three folds [9,10,11]. It can also increase the risk of falls, delirium, and hallucination [12,13]. Additionally, PIMs use is associated with a financial burden on the healthcare system [14].

The safety of population-specific medication use by older adults is getting increasing attention, specifically for geriatric psychiatry. There is growing evidence of the high prevalence of polypharmacy and potentially inappropriate medication use among older adults with anxiety disorder and depression [15]. For example, a study in Saudi Arabia that included 4187 elderly patients has reported that around 70% of older adults with anxiety disorder have potentially inappropriate prescriptions [16]. Polypharmacy among older adults with anxiety disorder is also common, with a prevalence of 75% [17]; consequently, older patients are at a higher risk of potentially inappropriate medications (PIMs) use.

Current evidence has many studies on PIMs use among older adults; still, few studies focus on PIMs use in geriatric psychiatry [18,19,20], which is getting growing attention. One study used information from encounters with general practitioners in Germany [20], and two other studies were among psychiatric inpatients and outpatients and not specific to older adults with an anxiety disorder [18,19]. The uniqueness of this research is the focus on older adults with anxiety disorders admitted to a large tertiary hospital and using electronic medical records, which allows us to obtain a sufficient sample size to do both descriptive and inferential analysis. Therefore, the purpose of this study is to determine the prevalence of PIM usage among older people who have anxiety and to investigate the variables associated with PIMs use in this population.

## 2. Methods

### 2.1. Design

In this cross-sectional retrospective study, twelve months (1 January 2021 to 30 December 2021) of data from the Electronic Health Record (EHR) database was used. Data of a tertiary teaching hospital in Riyadh, Saudi Arabia, were retrieved from the EHR database. The information was divided into files for demographics, clinical diagnoses, and prescription medications. The patient’s date of birth, gender, marital status, and nationality were all listed in the demographics file. The clinical diagnosis file contained data on the date of the clinical diagnostic and the clinical diagnosis from both inpatient and outpatient visits. Information regarding the prescribed medications, including type, dose, duration, frequency, route of administration, and quantity given, was recorded in the prescription medication file. Approval for this study was received from the Institutional Review Board (IRB) of King Saud University (reference number E-17-2580). Each participant gave their written, informed consent. To ensure confidentiality, health records for patients were kept on a password-protected computer with restricted access and no patient identifier.

### 2.2. Sample and Procedure

This study identified 371 older patients with anxiety disorder, aged 65 years and older, from ambulatory care clinics at a tertiary teaching hospital in Riyadh, Saudi Arabia, using twelve months of data retrieved from the EHR database. This study included older patients aged 65 years and older who have been diagnosed and who visited an ambulatory care setting with anxiety disorder using the IC-9-CM codes. We have excluded younger adults <65 years old and those from an inpatient setting.

### 2.3. Measures

#### 2.3.1. Dependent Variable: Potentially Inappropriate Medications (PIMs)

Estimating the prevalence of PIMs in older persons was the study’s primary goal. Two categories were utilized to classify the PIMs in accordance with the American Geriatric Society’s (AGS) updated 2019 Beers criteria: (1) drugs to avoid for the majority of older persons, and (2) medications to be used cautiously [21]. The prevalence of PIMs use was classified into (one PIM, two PIMs, and three or more PIMs). Then, PIMs use was classified into two groups: (1) PIMs use (i.e., use of one or more PIMs) and (2) non-PIMs use (i.e., no PIMs use). When more than one potentially inappropriate medication was administered to a subject throughout the research period, that subject was deemed to have been exposed to PIMs. We adapted all stated criteria that expert panels had previously provided for elderly people [21]; the criteria for “medications that should be avoided in the elderly” and “medications that should be used with caution in the elderly” were implemented entirely, except for the sections on drug–drug and drug–disease interactions.

#### 2.3.2. Independent Variables: Demographics and Co-Morbidity

Patient demographics, clinical information, and drug use were all used as independent factors. In terms of demographics, patient’s age in years, gender, marital status (single, married), and nationality (Saudi, Non-Saudi) are all included. Clinical information covered chronic illnesses such cancer, diabetes, dyslipidemia, hypertension, heart failure, ischemic heart disease, asthma, chronic obstructive pulmonary disease, osteoarthritis, and osteoporosis. ICD-9-CM (International Classification of Diseases, Ninth Edition, Clinical Modification) codes were used by doctors to report clinical diagnoses. The usage of five or more medications was referred to as polypharmacy. In the published literature, there are various ways to measure polypharmacy, including concurrent and cumulative methods [22]. Additionally, there is no consensus on the thresholds for how many prescription drugs should be considered to be regarded as polypharmacy. In the current study, polypharmacy was defined as the cumulative usage of five or more drugs over the course of a year; this cutoff point has been used more frequently than others.

### 2.4. Sample Size Calculation

The sample size was determined using an estimated prevalence of 11% and a margin of error of 4%. The program stated that the sample needed at least 236 patients to ensure the precision of the data. All 371 patients who met the eligibility criteria in 2021 were included in this study.

### 2.5. Statistical Analysis

For continuous variables, the mean and standard deviation (SD) were utilized, while for categorical variables, frequencies and percentages were employed to represent the study population. We performed a normality test to ascertain how the continuous data (i.e., age, number of medications, and number of chronic conditions) were distributed. The data were normally distributed; therefore, we performed a parametric test, the *t*-test, for age, number of medications, and number of chronic conditions. Pearson’s chi-square test was used for categorical variables (i.e., gender, marital status, nationality, all chronic diseases, and polypharmacy) to determine factors associated with PIMs use. After we carried out the regression assumptions, binary logistic regression analysis was used to examine the associations between PIMs use and the patient’s demographics, polypharmacy, and concurrent chronic health conditions. In logistic regression analysis, multiple regression models were used to evaluate the adjusted relationships between anxiety and PIMs use in which independent variables were entered in blocks. Model I evaluated the relationship between anxiety and PIM usage without accounting for any independent factors. Model II adjusted for demographic factors (age, gender, marital status, and place of residence). Model III adjusted for polypharmacy. Model IV adjusted for chronic conditions. To assess model fitness and model comparability, we employed Akaike’s Information Criterion (AIC). A 95% confidence interval (CI) and a significance threshold of =0.05 were taken into account.

## 3. Results

### 3.1. Study Sample Characteristics

The study sample includes 371 older adults (age ≥ 65 year) with anxiety disorder. The average age was (72.1 ± 5.8) years. The prevalence of hypertension, diabetes, and dyslipidemia was high in this sample: 58.0%, 45.6%, and 28.6%, respectively. Characteristics of the study sample are displayed in (Table 1).

### 3.2. Prevalence of PIMs

PIMs use among older adults with anxiety was (66.6%) (Table 1). About 35.6% used one PIMs, 22.6% used two PIMs, and 8.4% used three PIMs (Table 2). The most frequently prescribed PIMs were NSAIDs and gastrointestinal agents.

### 3.3. Factors Associated with PIMs Use from Unadjusted Bivariate Analysis

Older adults with anxiety who had coexisting chronic conditions, including hypertension, diabetes, heart failure, ischemic heart disease, and cancer, had significantly higher use of PIMs than those without these chronic conditions (*p*-value < 0.001). For example, older adults with anxiety and coexisting hypertension had a higher use of PIMS than those without hypertension (72.6% vs. 58.3%, *p*-value = 0.004). Additionally, polypharmacy was significantly associated with PIMs use compared to those without polypharmacy use (77.6% vs. 17.6%, *p*-value = 0.004).

### 3.4. Factors Associated with PIMs Use from Adjusted Regression Analysis

AIC was used to evaluate model fitness for model comparison. As a result, Model IV, which incorporated all of the factors with the lowest AIC value, was shown to offer the best fit. Four factors were associated with PIMs use in older adults with anxiety from the adjusted regression analysis; adjusted odds ratios (AOR) and 95% confidence intervals (CI) for factors associated with PIMs use are displayed in (Table 3). PIMs use was less likely among men compared to women. PIMs use was more likely among those with diabetes, cancer, and polypharmacy. Polypharmacy users were seven folds more likely to use PIMs than those without polypharmacy use (Table 3).

## 4. Discussion

The purpose of this study was to determine how frequently older patients with anxiety disorder used PIMs. Using the 2019 Beers criterion categories, two thirds of older adults with anxiety were found to have medications that should be avoided in the older population. Although the prevalence of PIMs was very high, it was still within the range of data from earlier research on Saudi older adults [16] but greater than that reported by Berger and colleagues [20], who found that 40% of elderly patients (65 years) with a diagnosis of generalized anxiety disorder use inappropriate medications.

The most frequently prescribed PIMs were NSAIDs, followed by gastrointestinal agents. Regarding older adults with anxiety disorder, the AGS Beers Criteria does not list specific recommendations regarding anxiety medications. However, it provides general guidance on certain medication classes commonly used for anxiety treatment, such as benzodiazepines, antipsychotics, and antidepressants. In our sample of 371 older adults with anxiety disorder, only 2.4% used benzodiazepines, 3.7% were on antipsychotics, and 12.2% used antidepressants that should be avoided in older adults. Using similar PIMs criteria, a cross-sectional study among older adults with a mental disorder reported that 7.8% had used PIM benzodiazepines and 22.4% used antipsychotics, and 1.2% used antidepressants that should be avoided in older adults [23]. Among older adults in general, potentially inappropriate use of benzodiazepines ranges from 9.3–12.9%; antipsychotics range from 1.8–8.3%; and antidepressants 2.2–2.3% [24,25,26,27]. It should be mentioned that benzodiazepines, in older adults, increase the risk of cognitive decline, delirium, falls, fractures, and car accidents [21]. Older adults are more sensitive to benzodiazepines and have a slower metabolism of long-acting drugs.

The most likely factor associated with PIMs use in this study was polypharmacy. We found that over two thirds of the study participants took more than five different medications. This finding is in line with the findings in the general population [16]. The higher rate of polypharmacy use in our study sample can be attributed to the higher rate of co-existing chronic conditions, for which they may need to take many medications to control their chronic conditions. Furthermore, co-existing diabetes and cancer in the study sample were important contributors to improper medicine use in this vulnerable population. According to studies, older persons with anxiety and other comorbidities have worse outcomes, require more intensive care, and have higher levels of psychological and social impairment than older adults with anxiety alone [28,29].

### 4.1. Study Implications

The above findings can be used to understand the elements that contribute to using PIMs better because it enables the evaluation of the elderly population’s need for new services aimed at them. Healthcare professionals’ roles should be expanded to ensure that the essential precautions are taken when managing older patients’ conditions to prevent incorrect medication prescribing. Additionally, the findings of this study give policymakers crucial information regarding the necessity of properly implementing pharmacy services, such as medication treatment management and continuous medication review on a regular basis, which have been shown to be advantageous by numerous studies [30,31,32]. For instance, ambulatory psychogeriatric patients with excessive polypharmacy benefit from pharmacist interventions to review medication use and minimize unnecessary prescribing [30]. Additionally, this study found that following clinical pharmacist recommendations reduced the total number of prescribed PIMs and the total number of medications used [30].

### 4.2. Strength and Limitations

This study has a few drawbacks. First, because this study was retrospective, some patient data needed to identify other categories of PIMs were not recorded in the EHR database. Second, because only older patients who visited ambulatory care clinics at one tertiary hospital were included in this study, the results cannot be generalized to all older persons in Saudi Arabia. Thirdly, this study did not fully assess the effects of other factors such as sociodemographic predictors, variation in clinical settings across the region, comorbidity index, or recent hospitalization, necessitating further research to fully evaluate these factors and the use of PIM among older adults with anxiety. Furthermore, to our knowledge, this is the first study to look at PIM usage in older patients with anxiety disorder and assess the predictors of PIM use in this vulnerable sample. As a result, this study provides greater insight into what is required to comprehend how to lessen the adverse effects of PIM misuse and improve care for older persons with anxiety to reduce health risks and financial burdens.

## 5. Conclusions

This study found that older people with anxiety disorders had a significant prevalence of PIMs that had to be avoided. In addition, chronic illnesses and polypharmacy were indicators of older patients’ greater usage of PIMs. Future research to investigate the adverse health outcomes related to PIM usage and measures used to justify using unneeded or high-risk drugs among this population are warranted, given the predicted rise of older people and the high prevalence of anxiety among them.

## Figures and Tables

**Table 1:** Characteristics of the study population. Number and raw percentage of characteristics by potentially inappropriate medication use.

		Total		PIMs Use		No PIMs Use			
		N	%	N	%	N	%	*p* Value	Sig.
Total		371	100	247	66.6	124	33.4		
Age Mean (SD)		72.1 (5.8)		73.3 (6.0)		72.1 (5.3)		0.069	
# Rx Mean (SD)		8.4 (4.2)		10.0 (3.7)		5.1 (2.9)		<0.0001	***
# Conditions Mean (SD)		2.8 (1.4)		3.1 (1.3)		2.4 (1.4)		<0.0001	***
Gender								0.017	*
	Male	207	55.8	127	61.4	80	38.6		
	Female	164	44.2	120	73.2	44	26.8		
Marital Status								0.321	
	Single	57	15.3	23	40.3	34	59.7		
	Married	314	84.6	154	49.1	160	50.9		
Nationality								0.539	
	Saudi	311	83.8	205	65.9	106	34.1		
	Non-Saudi	60	16.2	42	70.0	18	30.0		
Hypertension								0.004	**
	Yes	215	58.0	156	72.6	59	27.4		
Diabetes								<0.0001	***
	Yes	169	45.6	130	76.9	39	23.1		
Dyslipidemia								0.917	
	Yes	106	28.6	71	67.0	35	33.0		
Heart Failure								0.017	*
	Yes	21	5.7	19	90.5	2	9.5		
Ischemic Heart Disease								0.043	*
	Yes	22	5.9	19	86.4	3	13.6		
Asthma								0.990	
	Yes	36	9.7	24	66.7	12	33.3		
Osteoarthritis								0.345	
	Yes	21	5.7	12	57.1	9	42.9		
Osteoporosis								0.695	
	Yes	14	3.8	10	71.4	4	28.6		
Depression								0.380	
	Yes	6	1.6	5	83.3	1	16.7		
Cancer								<0.0001	***
	Yes	60	16.2	55	91.7	5	8.3		
Polypharmacy								<0.0001	***
	≥5	303	81.7	235	77.6	68	22.4		
	0 to 4 drugs	68	18.3	12	17.6	56	82.4		

Note: Data for 371 older adults, aged 65 years and older, with anxiety. *t*-test was used to assess the association between continuous variables and PIMs use. N: Number; PIMs: Potentially Inappropriate Medications; Rx: Medications; Sig: Significance; #: Number. Asterisks (*) represent significant differences in PIMs use from t-tests and Chi-square tests. *** *p*< 0.001; ** 0.001 ≤ *p* < 0.01; * 0.01 ≤ *p* < 0.05.

**Table 2:** Summary of the findings of potentially inappropriate medications to be avoided for most older adults according to the 2019 Beers criteria (n = 371).

		N	%
Average number of PIMs (SD)		1.07 (0.9)	
Average number of medications (SD)		8.4 (4.2)	
Potentially Inappropriate Medications Use			
	Yes	247	66.6
	No	124	33.4
Number of Potentially Inappropriate Medications			
	No PIM	124	33.4
	One PIM	132	35.6
	Two PIM	84	22.6
	Three or more PIM	31	8.4
Most common PIMs prescribed to be avoided			
	NSAIDs	240	64.6
	Gastrointestinal	188	50.67
	Cardiovascular	132	35.58
	Endocrine	45	12.13
	Antidepressants	45	12.16
	Antipsychotics	14	3.77
	Antispasmodics	9	2.43
	Benzodiazepines	9	2.43
	Anti-infective	3	0.81
	Genitourinary	1	0.27
	Antiparkinsonian agents	1	0.27
PIMs to be used with caution in older adults			
	Diuretics	112	30.19
	Antidepressant SSRI	29	7.82
	Vasodilators	17	4.58
	Antidepressant alpha-2 antagonist agents	2	1.89
	Anticoagulant agents	5	1.35
	Anti-neoplastic alkylating agents	5	1.35
	Anticonvulsant agents	1	0.27
	Anti-neoplastic agent, antimicrotubule	2	0.54

Data for 371 older adults, aged ≥65 years with anxiety. N: Number; NSAID: Nonsteroidal anti-inflammatory drugs; PIMs: Potentially Inappropriate Medications.

**Table 3:** Logistic regression on PIM use among older patients with anxiety.

		PIM Use		
		Adjusted Odds Ratio	95% CI	Sig.
Age Mean		1.02	[0.97–1.07]	
Gender				*
	Male	0.45	[0.25–0.85]	
	Female (Ref.)			
Marital Status				
	Single	0.67	[0.40–1.11]	
	Married (Ref.)			
Nationality				
	Saudi	1.31	[0.61–2.79]	
	Non-Saudi (Ref.)			
Hypertension				
	Yes	1.16	[0.62–2.1]	
Diabetes				**
	Yes	2.48	[1.39–4.43]	
Dyslipidemia				
	Yes	0.54	[0.26–1.08]	
Heart Failure				
	Yes	5.19	[0.86–31.3]	
Ischemic Heart Disease				
	Yes	3.48	[0.77–15.7]	
Asthma				
	Yes	0.57	[0.23–1.41]	
Osteoarthritis				
	Yes	0.5	[0.16–1.53]	
Osteoporosis				
	Yes	0.62	[0.15–2.46]	
Depression				
	Yes	4.17	[0.36–6.43]	
Cancer				**
	Yes	3.2	[2.59–9.3]	
Polypharmacy				
	≥5	7.7	[6.48–12.2]	***
	0 to 4 drugs (Ref.)			

PIMs: Potentially Inappropriate Medications; Ref: Reference group; Sig: Significance. Asterisks (*) represent significant differences in PIMs use, *** *p* < 0.001; ** 0.001 ≤ *p* < 0.01; * 0.01 ≤ *p* < 0.05.

## Data Availability

Due to our IRB policy, the EHR dataset utilized during and/or analyzed during the current study is not publicly available. However, the corresponding author can provide it upon reasonable request.
